# Complete Genome Sequence of Tsuneonella flava SS-21NJ, a Potential Oil Sludge Bioremediation Agent

**DOI:** 10.1128/MRA.00216-21

**Published:** 2021-05-20

**Authors:** Shuo Sun, Zhenhua Zhang, Cigang Yu, Yan Liu, Xian Xiao, Yuan Zhao

**Affiliations:** a School of Environmental and Safety Engineering, Changzhou University, Changzhou, China; b Key Laboratory of Biosafety, Nanjing Institute of Environmental Sciences, Ministry of Environmental Protection, Nanjing, China; University of Southern California

## Abstract

We report here the complete genome sequence of Tsuneonella flava strain SS-21NJ, which was isolated from oil sludge from Shengli Oilfield in Dongying, Shandong Province, China. These results provide basic information for functional genomics and oil degradation research of *Tsuneonella* strains.

## ANNOUNCEMENT

Here, we report the genomic data of the oil-degrading strain Tsuneonella flava SS-21NJ, which was isolated from oil-contaminated soil near the tidal flat in Shengli Oilfield in Shandong Province, China (37.55°N, 119.40°E). The oil-degrading strain was isolated according to the following steps: 1 g of oil-contaminated soil was added to 100 ml enrichment medium (mineral salt medium supplemented with 1% [wt/vol] crude oil); after incubation for 7 days, 1 ml of culture was transferred to 100 ml fresh enrichment medium. This enrichment procedure was repeated 3 times to obtain oil-degrading bacteria. The final 1-ml culture was diluted 10^−5^ to 10^−8^ and spread onto LB solid medium. After incubation at 33°C for 3 days, a single colony was transferred to and subcultured on LB solid medium until growth was obtained. A single yellow colony was isolated as strain SS-21NJ, grown on LB broth at 33°C for 24 h, and the genomic DNA was extracted using bacterial DNA kits (Omega, Norcross, GA) according to the manufacturer’s instructions.

In this project, the whole-genome shotgun (WGS) strategy was adopted to construct a library of different inserted fragments. Next-generation sequencing (NGS) technology was used, constructing a paired-end 250-bp DNA library based on the NovaSeq sequencing platform (Illumina, San Diego, CA, USA) and constructing a 20-kb DNA library based on the Sequel sequencing platform (PacBio, CA, USA). The libraries were size selected using the BluePippin system (Sage Scientific, Beverly, MA) with a cutoff size of 10 kb. For Illumina sequencing, the library was prepared using the TruSeq DNA sample prep kit. The pooled libraries were sequenced using the NextSeq system, yielding 7,717,940 reads with 1,960,521,844 bases that were trimmed using AdapterRemoval v2.2.2, which provided an average of 568× coverage. For PacBio sequencing, the library was prepared using the PacBio template prep kit 1.0, yielding 73,418 reads (*N*_50_, 9.1 kbp) and a minimum DNA size of 200 bp. The PacBio sequencing yielded 73,418 reads with 569,497,913 bases. The PacBio data were assembled using HGAP version 4 (1) in SMRT Analysis software version 2.3.0 (Pacific Biosciences) (2), and another assembly was performed using CANU version 1.7.1 (3) software, using the complete set of subreads from the single-molecule real-time (SMRT) cell. The HGAP assembly was used for the final assembly, with the overlapping ends removed using HGAP, to obtain a circular genome sequence. The PacBio sequences were checked with the Illumina sequences and corrected using unicycler_polish with Pilon version 1.18 (4, 5) with default settings.

The genome of strain SS-21NJ is composed of a single circular chromosome of 3,284,110 bp, with a mean G+C content of 60.55%. No plasmids were identified in the genome. Genome annotation was performed on the NCBI Prokaryotic Genome Annotation Pipeline (PGAP) version 4.13 (https://www.ncbi.nlm.nih.gov/genome/annotation_prok/). A total of 3,062 genes were predicted by PGAP, including 2,970 protein-coding genes. The genome encodes 47 tRNAs and 3 rRNAs, which includes 1 copy each of the 5S, 16S, and 23S rRNA genes. The details of the sequencing reads can be found in Table 1. The 16S rRNA gene sequence of strain SS-21NJ showed 99.86% similarity to that of *Tsuneonella flava* M1-4^T^ (GenBank accession number GCA_002870965.1), according to a BLASTn search using the 16S rRNA gene sequence of strain SS-21NJ as a query and using strain MS1-4 as a reference. Default parameters were used for all software unless otherwise specified.

**TABLE 1:** Summary of the general genomic features of *Tsuneonella flava* strain SS-21NJ

Item	Statistic
Chromosome size (bp)	3,284,110
G+C content (mol%)	60.55
No. of genes No. of protein-coding genes No. of tRNA genes	3,062 2,970 47
No. of 5S rRNA genes	1
No. of 16S rRNA genes	1
No. of 23S rRNA genes	1

### Data availability.

The complete genome sequence of *Tsuneonella flava* SS-21NJ was deposited in the GenBank database under accession number CP061510, where the PGAP annotation is also available. The reads were deposited in the Sequence Read Archive (SRA) under BioProject accession number PRJNA663285 and BioSample accession number SAMN16125023.
